# The immune effects of mesenchymal stem cell-based therapy in kidney diseases

**DOI:** 10.1093/lifemedi/lnag008

**Published:** 2026-04-07

**Authors:** Yachao Li, Kuai Ma, Junyi Ren, Haoyu Peng, Moussa Ide Nasser, Chi Liu

**Affiliations:** School of Medicine and Life Sciences, Chengdu University of Traditional Chinese Medicine, Chengdu 610041, China; Department of Nephrology, Osaka University Graduate School of Medicine, Osaka 113-8654, Japan; School of Medicine, University of Electronic Science and Technology of China, Chengdu 610041, China; School of Medicine, University of Electronic Science and Technology of China, Chengdu 610041, China; Department of Cardiac Surgery, Guangdong Provincial People’s Hospital (Guangdong Academy of Medical Sciences), Southern Medical University, Guangdong Cardiovascular Institute, Guangzhou 510100, China; Department of Nephrology, Sichuan Clinical Research Center for Kidney Disease, Sichuan Provincial People’s Hospital, University of Electronic Science and Technology of China, Chengdu 610041, China

Kidney disease remains a major global health issue, characterized by elevated morbidity and mortality rates. Current interventions encompass pharmacological treatments, hemodialysis, peritoneal dialysis, and renal transplantation [1]. However, the efficacy of these interventions is inconsistent, primarily due to the irreversible nature of kidney damage, the potential for severe drug-induced side effects, variability in dialysis outcomes, and the persistent shortage of donor organs [1]. In the context of kidney disease, a self-perpetuating cycle of inflammation, immune cell infiltration, and cellular apoptosis results in the deposition of fibrous matrices, which disrupt the structural integrity of renal tissue and progressively impair kidney function [2]. Renal immunity is orchestrated by a variety of innate immune system cells, including dendritic cells (DCs), macrophages/monocytes, natural killer (NK) cells, T cells, B cells, and neutrophils [2].

**Figure 1.**
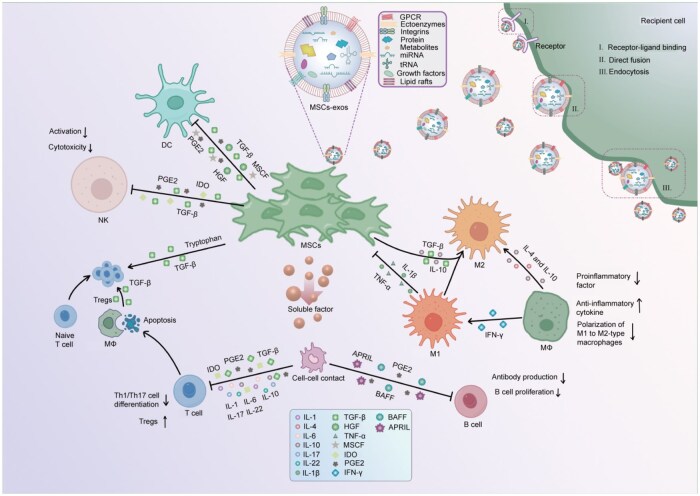
Immunomodulatory mechanisms of MSCs via soluble factors and intercellular crosstalk.MSCs regulate immune responses by releasing soluble factors and extracellular vesicles. Furthermore, through the secretion of ­molecules such as indoleamine 2,3-dioxygenase (IDO), prostaglandin E2 (PGE2), and transforming growth factor-β (TGF-β), MSCs suppress the differentiation of pro-inflammatory Th1 and Th17 cells and promote the expansion of regulatory T cells (Tregs). Additionally, MSCs also limit B-cell proliferation and antibody production via soluble mediators and cell–cell interactions involving BAFF and APRIL. Likewise, in the innate immune system, MSCs dampen inflammation by reducing macrophage activation and promoting a shift from the pro-inflammatory M1 phenotype to the anti-inflammatory M2 phenotype, which is associated with decreased pro-inflammatory mediators and increased IL-10 production. MSCs inhibit natural killer (NK) cell activation and cytotoxicity through IDO and PGE2. MSCs inhibit dendritic cell (DC) through TGF-β, PGE2, MSCF, and HGF. Also, MSCs inhibit natural killer (NK) cell activation and cytotoxicity through IDO and PGE2. Finally, MSC-exos further contribute to immune regulation by transferring bioactive molecules to recipient cells via receptor binding, membrane fusion, or endocytosis.

Mesenchymal stem cells (MSCs) and their paracrine pro­ducts, including mesenchymal stem cell–conditioned medium (MSC-CM) and MSC-derived exosomes (MSC-exos), demonstrate various therapeutic properties, primarily via immunomodulatory mechanisms that regulate the equilibrium between effector T cells and regulatory T cells (Tregs), inhibit B-cell activity, and adjust the functions of innate and adaptive immune cells (Fig. 1).

Numerous studies have shown that MSC-based therapies have emerged as promising treatments for a variety of diseases, including renal diseases. Their therapeutic mechanisms are primarily characterized by broad immunomodulatory effects, which include the regulation of the balance between effector T cells and regulatory T cells (Tregs), suppression of B-cell activation and antibody production, polarization of pro-inflammatory M1 macrophages toward the anti-inflammatory M2 phenotype, and inhibition of dendritic cell (DC) activation and natural killer (NK) cell proliferation and cytotoxic activity (Fig. 1). MSCs and their paracrine products contribute to the preservation of renal structure and function by attenuating renal inflammatory responses, also by limiting immune-mediated tissue injury, and affecting both innate and adaptive immune cells through these coordinated actions [1].

Recent studies have demonstrated that human mesenchymal stem cells (hMSCs) can modulate immune responses in glomerulonephritis (GN) by influencing T cell subsets. Specifically, hMSCs inhibit renal cortical mRNA expression of IL-17 and serum IL-17A levels, while upregulating *FOXP3* mRNA, a transcription factor specific to Tregs. MSCs can suppress the Th1 and Th17 response and induce the Th2. This suggests that hMSCs interact with Th1, Th2, Th17, and Tregs in the kidney or other immune organs to exert immunomodulatory effects. For example, MSC treatment in rats has been shown to reduce intra-glomerular CD8^+^ T cell infiltration [3]. MSCs-based therapy has also demonstrated immune regulatory effects in a clinical trial. MSC-based therapy has also shown immunoregulatory effects in a clinical trial. Indeed, in a Phase I clinical trial (CT 2016-004804-77) involving pediatric patients with renal disease, the administration of bone marrow mesenchymal stromal cells (BMSCs) induced a significant but transient reduction in total B cells, mature B cells, and memory B cells. The immunosuppressive therapy was gradually tapered, and B-cell populations progressively recovered, which was accompanied by an increase in memory-activating regulatory T cells (Tregs) [4]. In a fatal Lupus nephritis (LN) model utilizing NZB/WF1 (BWF1) mice, human embryonic stem cell-derived mesenchymal stem cells (hESC-MSCs) were found to significantly reduce proteinuria and serum creatinine levels, preserve renal architecture, and markedly decrease serum concentrations of TNF-α and IL-6. Mechanistically, *in vitro* experiments support these findings: co-culturing hESC-MSCs with lipopolysaccharide (LPS)-stimulated BWF1 lymphocytes led to a reduction in lymphocyte secretion of TNF-α and IL-6, along with an increase in the proportion of Tregs [5]. Not only that, but a large number of experiments have also proved that MSC-based treatment is widely used in the treatment of kidney diseases such as acute kidney injury and chronic renal failure.

In summary, MSCs are highly accessible and exhibit multifaceted mechanisms of action, making them a promising therapeutic option for a wide range of diseases. Their secreted products, including MSC-exos and MSC-CM, have been widely employed in the treatment of various preclinical models, particularly renal disorders, owing to their low immunogenicity and high biocompatibility. However, several future research directions for MSC-based therapies warrant attention. First, although MSCs are widely utilized in clinical therapies due to their immunomodulatory effects, the underlying mechanisms of action remain incompletely understood. Further investigation into these mechanisms is essential to optimize their therapeutic potential. Second, MSCs are derived from a variety of tissue sources, and clinical practice currently employs MSCs from multiple origins. To enhance the therapeutic efficacy of MSCs, it is crucial to explore and develop MSCs from additional sources, expanding the scope of their clinical applications. Third, due to the inherent heterogeneity of MSCs, there is a growing need to advance personalized MSC-based therapies. Tailoring MSC treatments to individual patient characteristics could improve therapeutic outcomes and minimize variability in clinical responses. Lastly, experiments investigating the use of MSCs in the treatment of pulmonary fibrosis have shown that MSCs can secrete TGF-β to modulate immune responses, thereby alleviating inflammation. However, MSCs can also act as targets of TGF-β, leading to increased collagen expression and deposition, which suggests that under certain conditions, they may shift from inhibiting fibrosis to promoting it. In addition, MSC-exos offer advantages such as low immunogenicity, low cytotoxicity, and low mutagenic potential. Nevertheless, factors including the tissue source of MSCs, variations in culture conditions (e.g. nutrient composition and oxygen tension), and storage parameters (e.g. temperature and duration) directly influence the structural integrity and functional stability of exosomes. Therefore, standardized production processes are essential to reduce risks associated with MSC-based therapy, improve safety profiles, and support broader clinical translation. Large-scale clinical trials will be required to evaluate their efficacy and safety further.

Extensive research confirms the efficacy of MSCs in kidney diseases, primarily through anti-inflammatory effects, promotion of tubular regeneration, and immunomodulation [1]. Key future directions include: elucidating unresolved mechanisms of action; exploring novel MSC tissue sources; advancing personalized MSC therapies; and conducting large-scale clinical trials to enhance safety and clinical translation. Optimizing delivery ­methods, homing efficiency, and investigating synergistic combinations (e.g. with pharmacotherapy or gene editing) are critical for establishing MSC-based therapies in immune-mediated kidney diseases.
